# Anti-Conservative Treatment of Exposed Dental Pulp

**Published:** 1892-11

**Authors:** Charles Harker


### ANTI-CONSERVATIVE TREATMENT OF EXPOSED DENTAL PULP.[1]

BY CHARLES HARKER, D.D.S.

THE title of my paper makes clear the fact that, with me, the method of treating exposed pulps is the radical one. To this method I was driven by my almost universal failure in pulp-capping and palliative treatment previous to the last five years.

During this period I have devitalized more than ninety-five per cent. of all exposures presenting in practice, with no per cent., so far as I know, of failures to record.

If, therefore, I have been successful in any department of practice, it is with these most difficult and perplexing cases; and if I can convey intelligently this anti-conservative method to one young operator, sparing him and his patients my early failures, I shall feel more than repaid for my endeavors.

I say most difficult and perplexing, although, if we read over the articles on conservative treatment which have appeared in dental journals during the last decade, we might conclude that the dental pulp is the most manageable and accommodating of vital tissues.

I am well aware that I shall be arraigned by the able conserva-

---

[1] Abstract of a paper read at the joint meeting of the Pennsylvania and New Jersey State Dental Societies, July 20, 21, 22, 1892.

tive advocates present, for my so-called wholesale destruction of vital organs; but, be this as it may, I must state my convictions, based as they are upon my own experience.

When I state that I devitalize ninety-five per cent. of all exposed pulps, with no per cent. of failures, I do not wish to be understood as claiming immunity from trouble subsequent to devitalization, extirpation, and root-filling. Teeth having abnormally-formed or compressed roots, constrictions, nodules of dentine in canals, pulp-stones, etc., present difficulties not insurmountable, but requiring skill and patience for their successful treatment.

In my practice, in less than one per cent. of the teeth operated upon has irritation occurred of a nature serious enough to require removal of the permanent filling; and in these exceptional cases subsequent treatment has resulted in restoration to comfort and usefulness. In no one do I recollect an abscess having formed subsequent to root-filling, followed by extirpation of the pulp.

On the other hand, my experience in capping the fully-exposed pulp, due to extensive caries, has been almost always followed by pain to the patient and trouble to myself or my neighbor-dentist, with disaster to the tooth within two years unless the pulp was promptly devitalized, removed, and the root-canal properly filled. I say disaster to the tooth, because when the tooth is left to absorb the putrescent products of a decomposed pulp, the tissue itself suffers irreparable injury, not only in appearance but structurally as well. The dental tubuli having become saturated with the gaseous and sanious products of decomposition, the basal structure altered, the enamel discolored, checked, and brittle, a tooth is presented unwholesome to its surroundings and infinitely inferior in strength and comeliness to the one where the exposed pulp has been extirpated at sight or devitalized by the usual process, extracted, and the subsequent operations of root- and crown-filling completed properly and at the right time. The strength, color, or structure of the tooth is but slightly altered.

I have two teeth in my own mouth the pulps of which were devitalized, the roots filled with creosoted cotton, the crowns with amalgam, about twenty-two years ago, by my preceptor, Dr. Brown. After the lapse of twelve years the fillings were removed and the cavities refilled; until this time I did not know the pulps had been devitalized, and, with the exception of a little peridental irritation of one at the time, due to the unnecessary removal of root-filling to substitute a solid one at the apex (in most cases a bad practice), the teeth and surroundings have been perfectly healthful and useful.

They appear to be good for at least a score more years of active service.

I could give instances of teeth having been treated likewise, in which more than forty years of uninterrupted health and usefulness have been enjoyed.

Notwithstanding cases similar to these frequently come under our observation, I do not claim that pulps should never be capped. In traumatic cases, where the excavator has accidentally punctured the peridental membrane, or where fracture has occurred, slightly exposing the pulp, capping should be practised; especially as cases of this nature are usually the most favorable.

No one, I think, is more careful than the speaker about lining deep cavities in teeth whose pulps are not exposed. Magitot, in his treatise on dental caries, not only tells us, but clearly demonstrates, by beautifully executed cuts, the pulp complications where no exposure, or even near approach to it, exists.

Capping with oxyphosphate or oxychloride, to which is added about one part of carbolated vaseline to ten parts of the oxide, I believe conduces very materially to conservation of the pulp's vitality; and yet, more or less frequently, we all have pulps to die when capped under these most favorable circumstances.

Whether the loss of vitality is to be attributed to the continued irritation due to previous caries, the impingement of secondary dentine, thermal changes, the taking of cold, or what not, the result is the same; proving that, instead of being easily manageable, a tooth-pulp is so treacherous that we can never predict with certainty the results of capping.

This operation is based upon a most beautiful theory: there are many beautiful theories that do not work well in practice. I have always been in the habit (it may be a bad one) of uncapping all capped pulps coming from the hands of other dentists; that is, when refilling has become necessary, or disease is apparent. In this way I gain certain knowledge of the conditions present at the time of my operation which, it seems to me, can be gained in no other way. In almost every case in which the tooth has remained comfortable I find no real exposure exists nor ever did exist.

A small per cent. of these, however, have quietly succumbed, especially those in which the regular oxy-phosphate mixture has been used. Cases in which real exposure existed at the time of capping I have usually, although not always, found dead. When alive they appear, as they did before the capping, more or less diseased, but apparently resisting progressive diseased action while the vital forces are strong in the individual, but ready, whenever systemic changes occur reducing vital force, to develop a local inflammatory condition, resulting in death.

I am aware those who advocate conservative treatment claim, by properly-directed antiphlogistic measures, to be able to allay all irritability; but to my mind the treatment of an inflamed tooth-pulp by leeching, cold application, purges, etc., while the irritant (the plug) remains *in situ*, is too absurd to need comment.

I have yet to find the first instance of a previously exposed pulp due to caries having itself after capping wrought out physiologically the theory which the capping process promises, or rather, I should say, the result which the conservative advocate promises for the theory. That this physiological deposit ever takes place after caries has caused a rupture of the pulp-membrane, is still a question. No one, I think, would assert that the pulp ever does return to a perfectly healthful normal condition. That it may do so after exposure due to traumatic cause, is more reasonable to suppose.

In my experience, the membrane when ruptured by caries does not heal at all; when uncapped, it always presents a raw surface. I have, however, in a number of instances accidentally punctured the pulp in excavating, and, having capped and filled with cement, after the lapse of one year or more, upon uncapping I have found no exposure existed, calcified matter having formed under my cap. Underneath the calcified deposit I have no doubt cicatricial tissue had formed.

In the case of a girl, ten years of age, who had, by a fall, broken off the mesial corner of a superior central incisor, exposing pulp, so as to bleed slightly, I at once capped and built out to the original contour with gold. Five years afterwards, while playing at school, she again fell, breaking away the entire filling. Upon removing the cap, I found this pulp beautifully protected by a secondary deposit of well-formed tooth-bone.

This case, though interesting to study, does not come under conservative treatment in the usual acceptation of the term. I merely cite it to show the distinction, and also to show that we must have the ruptured membrane re-formed before we can have its function. Experience proves that after rupture by caries it does not re-form; it cannot, therefore, produce the secondary deposit claimed by the conservative advocate.

Since this paper has been in the course of preparation, one of these patients has visited my office, in whose lower jaw I found six dead teeth, which had been well and scientifically capped by an eminent Philadelphia dentist. Another had been treated in whose mouth I found three suppurating abscesses, with another tooth in the incipient stage, the results of well-capped pulps. I might enumerate cases which are striking illustrations of disaster resulting from unwise but well-meaning endeavors at pulp-conservation on the part of otherwise successful practitioners.

I do not believe with some that the whole office of a tooth-pulp has been performed when it has finished the construction of the tooth in which it is located; on the contrary, I hold that its function never ceases while it is in comparative health.

It continues to nourish the dentine, and under some circumstances to elaborate secondary dentine, notably when the pulp is being encroached upon, owing to mechanical or chemico-mechanical abrasion, and when caries is threatening exposure. I do not, therefore, wish, nor would I dare to claim, that a dead tooth is as good as a live one, other things being equal. I believe I practise conservative treatment of teeth by non-conservative treatment of the diseased pulp.

Is this not good surgery? In the practice of general surgery, does the surgeon hesitate to destroy the diseased part of an organ, if thereby the function of the whole may be preserved? Should we, then, as special surgeons, hesitate, especially as we, unlike them, are able to substitute artificially the lost part so perfectly that our patient feels, sees, or knows no loss?

The operations involved in dealing with exposed pulps, whether by extirpation or capping, call for skill and nicety; careless or indifferent operations will fail in the former sometimes, in the latter always.

Indeed, upon the exercise of judgment and skill in this department depends very largely the number of our patients; it is vital to our success. How, then, shall we treat them? Two methods are open,—palliative and radical. Upon the former I have dwelt sufficiently long; the latter I will now endeavor briefly to describe as I practise it. There may be nothing new or superior in it, but when thoroughly performed it is successful.

The teeth do not to any extent check, chip, break, discolor, or give other annoyance to patient or dentist. The models which I will pass may assist me in showing the partial and completed operations.

The plug is removed, or, if a fresh exposure, place over the exposed portion of the pulp pure carbolic acid for about two minutes, then, covering with sandarac and cotton, dismiss until the next day; at which sitting the previous medicament is removed, and the arsenious acid applied directly to the exposure, sealing again with sandarac and cotton.

I request the patient to call on the fourth day, when the arsenic is removed, the pulp entirely uncovered, and a portion excised, bleeding it freely to prevent pinking of tooth during the waiting time, about three weeks, while nature is deciding how much of the pulp she will give up. I then place in the pulp-chamber a piece of dry absorbent cotton, carrying as much as it will of iodoform powder, the piece of cotton being large enough to fill the pulp-chamber.

The cavity is then filled with a temporary gutta-percha stopping until the final operation, when I place dam upon the tooth, and extract pulp from root or roots. With No. 7 or 8 uncut Swiss broach having a perfect taper point I work a mixture of campho-phenique, iodoform, and zinc oxide to apex, following with a tiny fibre of cotton, carried as nearly to the point as the previous mixture will allow without painful pressure.

This procedure insures carrying a bland sterilizing apical stopping entirely to the so-called "apical space,"—that is, to the borderline separating devitalized dentine from living cementum and its investing membrane.

Removing with a broach the fibre of cotton, and drying the canal as thoroughly as possible, the remainder of the canal, when of normal calibre and formation, is filled with a gutta-percha cone made to fit the canal, the point of the cone having been moistened with chloroform previous to introduction.

The apical portion in two-rooted bicuspids, compressed anterior root of lower molars, and other tortuous or constricted canals, may, by using No. 7 slender Swiss broaches, usually be well filled either with the antiseptic material I have indicated or with chloro-gutta-percha.

Except for the removal of adherent and other calcific deposits, I never ream canals more than may be done with ordinary bud-shaped excavating burrs; canals which may not be reamed without great danger of going through the side of the root or breaking off the reamer, may be well filled without reaming. Nicely-tempered slender broaches adapt themselves without danger of breaking; reamers do not.

There is a field for the exercise of judgment in removing or overcoming the occasional difficulties of the apical territory, which perhaps no written description would cover, but which the cultivated judgment and touch, together with delicately-formed and properly-tempered instruments, will enable us to surmount with comparative ease.

Some of these exceptional conditions would have caused trouble, and ultimately death, had the pulp been capped and the capping operation itself been successful.

I do not remember ever having heard mentioned the method of approach which I use in dental exposures of lower molars, and would call attention to it, knowing from practical experience that by cutting away the posterior buccal corner as far as the buccal seam, instead of the usual drilling of a hole through the buccal wall, our light, line of vision, directness of approach, and movements of instruments are less obstructed.

I think you will also agree with me that, in addition to facilitating the whole operation, a tooth thus prepared is stronger than when filled in the usual way.

Let me say that if the arsenic has been well triturated, the proper dose used, the nerve well exposed before applying, the sandarac bottle warmed before using, and no pressure made upon the pulp in sealing up, there will very rarely be any pain from the application, and no danger from the effect of arsenic upon external peridental membrane, either about the apex or gingival margin.

In case the effect has not reached through the whole extent of the pulp, I remove the portion devitalized, re-apply a minute quantity of paste,—one part arsenious acid to ten parts iodoform, moistened with campho-phenique,—or anæsthetize locally with cocaine, and remove.

I much prefer removing the entire pulp to the apical constriction, but, if unable to do so, no harm will come from leaving in the canal the slender apical fifth of pulp, whether dead or alive, provided the antiseptic filling I have recommended be well picked into it; if not dead, it will die, and make a fairly good apical filling. When trouble follows devitalization, it is from one of two causes,—either peridental irritation arising from an overdose of arsenic, or ultimate disintegration and evolution into septic gases of unsterilized, shred-like portions of the pulp remaining in the inaccessible portions of tortuous or constricted root-canals. In my experience the former difficulty rarely occurs, and always subsides in a few days without treatment. I have already referred to the infrequency of the latter; when it does occur it is of more serious nature, but its consideration would take us into the treatment of incipient abscess, a discussion beyond the legitimate compass of my paper.

